# Topical Wharton's Jelly MSC‐Derived Age Zero™ Exosome Treatments After Micro‐Needling for Skin Rejuvenation

**DOI:** 10.1111/jocd.16561

**Published:** 2024-10-04

**Authors:** Corine Cicchetti, Carla Mazzeo, Michael Heke, Michael Crowley, Akis Ntonos, Erin Crowley

**Affiliations:** ^1^ Resiliélle Cosmetics™, LLC USA; ^2^ Regenerelle®, LLC USA; ^3^ Buffalo Regenerative Medicine USA; ^4^ The Crowley Center for Regenerative Biotherapeutics LLC USA; ^5^ Aion Aesthetics USA

To the editor,

The cosmetic industry is nowadays focusing more on natural products due to the increased awareness of the risks associated with using chemicals in cosmetics [1]. The global market for rejuvenation products is predicted to expand at a compound annual growth rate (CAGR) of about 6.7% from 2022 to 2027. As of 2021, the market size amounted to roughly 63 billion US dollars, projected to reach 93 billion US dollars by 2027 (*Source: Statista*). A dominant trend is identifying a natural, truly efficacious, and safe source for rejuvenation which will propel the projected market increase in future years. Exosomes are a natural source; they are tiny particles secreted by most cells in the body to facilitate cell‐to‐cell communication [2]. They carry proteins, lipids, and genetic material to other cells to reprogram and/or facilitate their optimal functionality. To evaluate the feasibility and effectiveness of exosome treatments for improving skin health, a study was performed to evaluate the potential benefits of Age Zero™ exosomes as previously proposed to achieve skin rejuvenation, wound healing, anti‐inflammation, and anti‐aging [3].

Exosomes derived from human umbilical cord Wharton's jelly mesenchymal stromal/stem cells (hUCWJ‐MSCs) are the best source for cosmetic purposes, as WJ‐MSCs have a high differentiation potential, an immune‐privileged status, are easy to collect and deliver exosomes with the most enriched information for renewal and regeneration [4]. A study is being conducted to evaluate the efficacy of Regenerelle's manufactured Wharton's jelly MSC‐derived “Age Zero™” medical grade “low‐passage” exosome products, which were applied topically after micro‐needling to treat age‐related skin conditions and to rejuvenate the skin.

Participants of this study benefited from accelerated redness reduction and healing after micro‐needling and showed improvements in skin texture, tone, pore size, fine lines, wrinkles, pigmentation, and acne scarring. Exosome treatments also lead to increased fibroblast proliferation and collagen synthesis, which are essential for healthy skin and tend to decrease with age [5]. Overall, the treatment showed results in younger looking and feeling skin.

The study involved the use of hUCWJ‐MSC‐derived exosomes. These Age Zero™ exosomes, suspended in physiological, aseptic saline solution, were administered topically after micro‐needling, provided by the trial's sponsor, *Resiliélle LLC*, at a concentration of 5 billion Age Zero™ exosomes in 2.5 mL saline. The SkinPen Precision System was used for micro‐needling [6] and the study included over 100 participants.

The treatment protocol defined a total of three treatments in 4‐ to 6‐week intervals and involved a standard micro‐needling treatment and the subsequent topical application of exosomes. Before each procedure, pictures were taken from various angles, with final photographs taken 4–6 weeks after the last treatment as a follow‐up. The study spanned over roughly 6 months, with each visit taking about 60 min for the first three treatments and about 15 min for the final assessment.

After every appointment, the participant was given a questionnaire to evaluate their experience with the procedure and to identify any subjective benefits. The questionnaire asked whether the participant noticed any improvements in wrinkles and fine lines, skin texture, skin firmness, spots and pigmentation, radiance, visible pores, moisture, and redness. The results were recorded as either YES or NO.

Pictures taken and self‐assessment questionnaires filled out by the participants were shared with the sponsor for analysis.

This study suggests that the use of Wharton's jelly “Age Zero™” MSCs as powerful source of rejuvenating exosomes that can be used in cosmetics to address age‐related skin conditions. This approach provides a safe, high‐quality, natural, and healthy way to restore the skin by delivering a range of specific components that are “designed” to, and have the innate ability to reprogram cells, resulting in a more youthful appearance (Table 1 and Figure 1).

**TABLE 1:** Improvement in skin appearance. The table displays the results of a 6‐month study on 100 participants who underwent a 5 billion Age Zero™ exosomes treatment after micro‐needling. The treatment was administered three times at 4‐ to –6‐week intervals. The study evaluated participants' appearance based on various criteria, including wrinkles and fine lines, skin texture, skin firmness, spot and pigmentation, radiance, visible pores, moisture, and faster redness reduction after MN.

Appearance	Improvement (%)
Wrinkles and fine lines	86.4
Skin texture	90.3
Skin firmness	88.0
Spot and pigments	88.9
Radiance	92.6
Visible pores	79.2
Moisture	95.0
Redness	85.0

**FIGURE 1:**
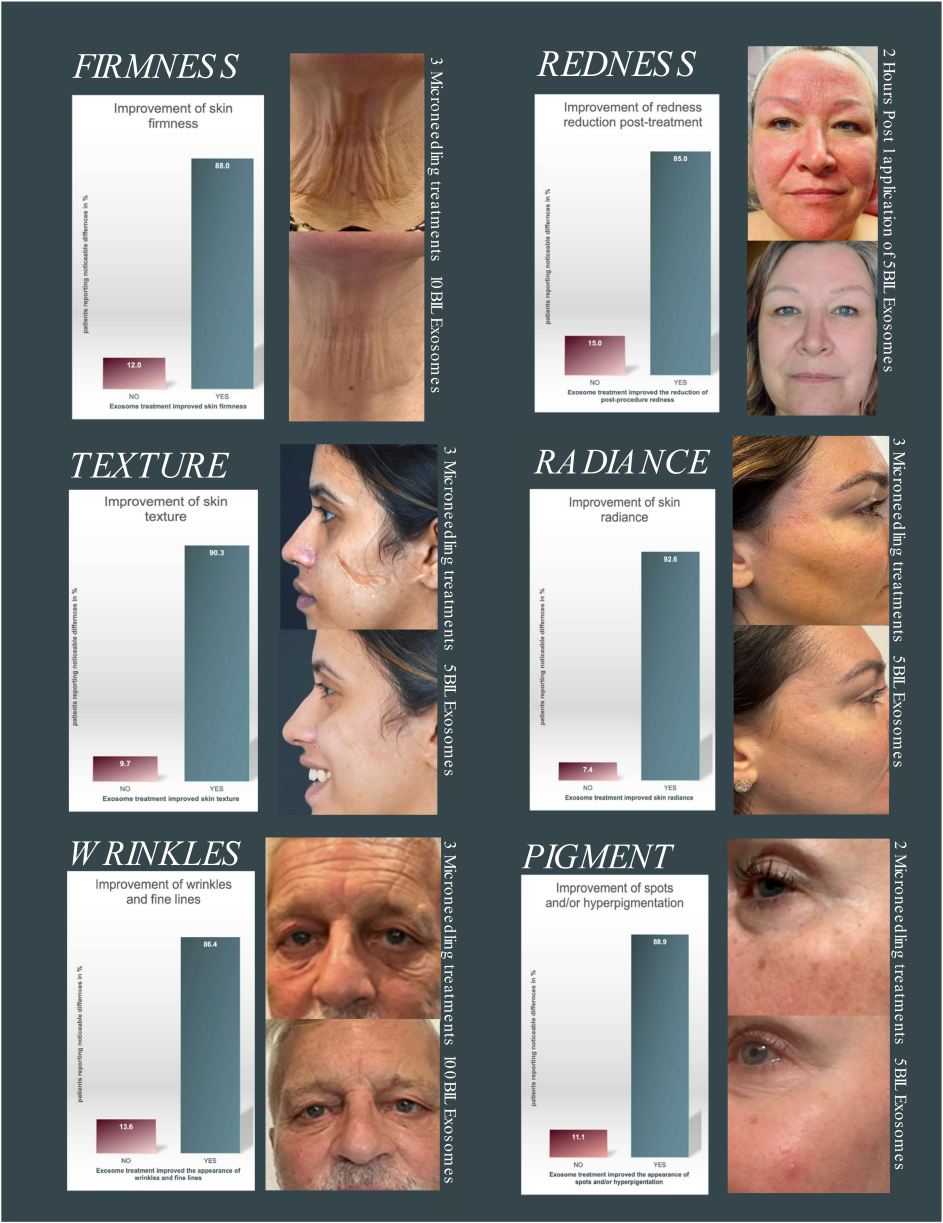
Improvement after Age Zero™ exosomes treatment. The graphs that correlate with the participant's pictures show the participant's noticeable difference in the skin appearance (%) with YES/NO answers derived from a questionnaire that asked to evaluate wrinkles and fine lines, skin texture, skin firmness, spots and pigmentation, radiance, visible pores, moisture, and redness. Pictures were taken before the first treatment, before each subsequent treatment, and final pictures were taken 4–6 weeks after the third and last micro‐needling treatment.

## Author Contributions

**Corine Cicchetti:** study design and execution. **Carla Mazzeo:** preparation of the manuscript. **Michael Heke:** study design, analysis of results, and preparation of figures. **Michael Crowley** and **Erin Crowley:** study design, analysis, and study supervision. **Akis Ntonos:** study design and execution. All authors read and approved the final version of the manuscript.

## Ethics Statement

The authors have nothing to report.

## Consent

Consent/Permission to be in the Research.

## Conflicts of Interest

Dr. Corine Cicchetti is the Chief Medical Officer of Regenerelle and Resiliélle has consultative or financial relationships with several of study the investigators may have with the BRMA and affiliates.

## Supporting information


Data S1.

## Data Availability

The data that support the findings of this study are openly available in Supporting Information/Figure 1/Table 1 at https://figshare.com/s/0139ddd82f28f5458a54.
