# Supplementary material for: Topical Wharton's Jelly MSC‐Derived Age Zero™ Exosome Treatments After Micro‐Needling for Skin Rejuvenation
Source: J Cosmet Dermatol. 2024 Oct 4;23(12):4389–91. doi: 10.1111/jocd.16561 (PMC11626297; doi:10.1111/jocd.16561)

**Supporting Information:**

- Individual graphs

- Questionnaires


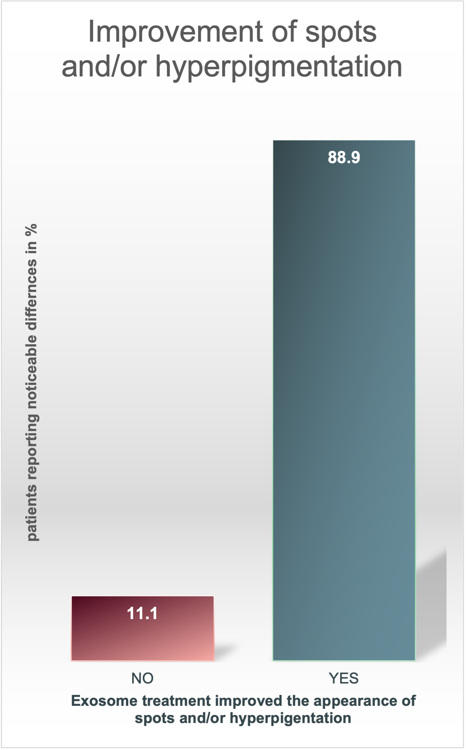

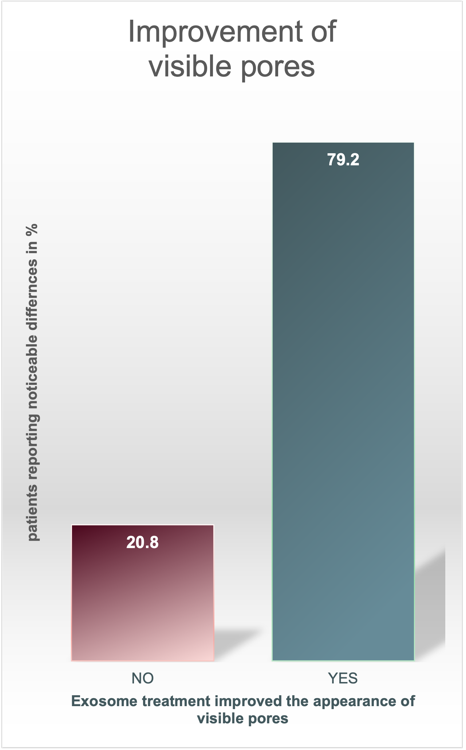


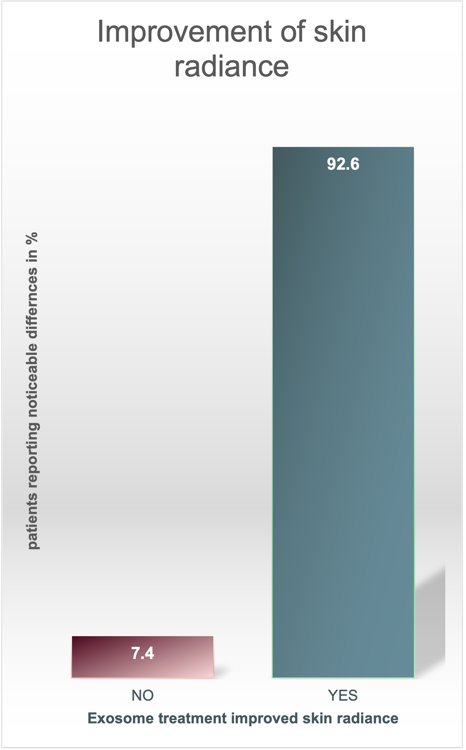

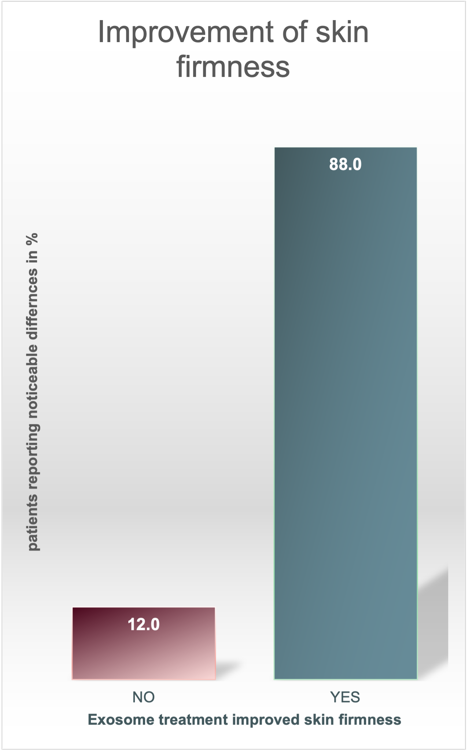


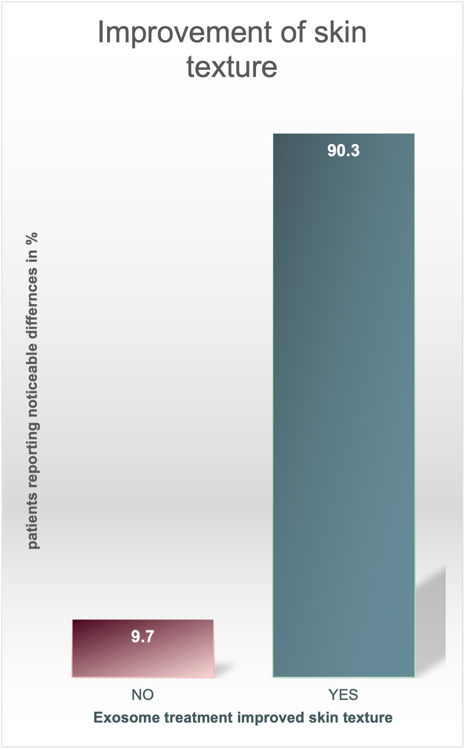

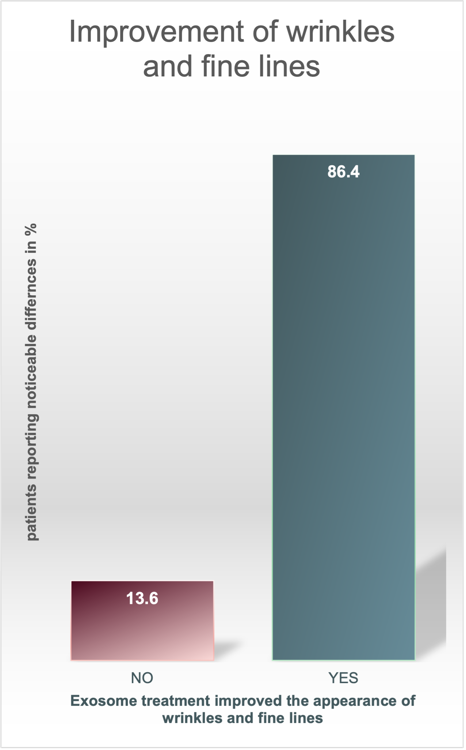


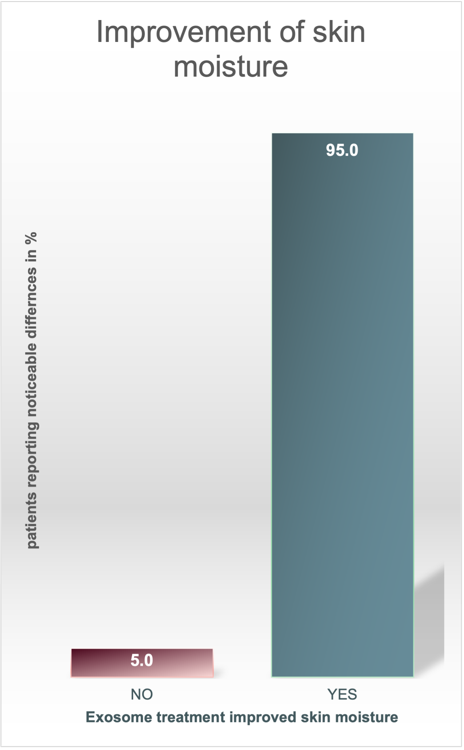

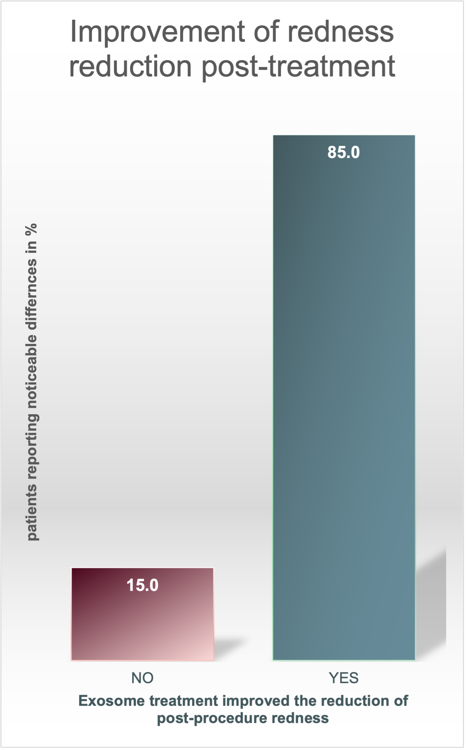


**Questionnaires:**

Initial Questionnaire:

<https://forms.gle/nxMF5SKzQ9HvwaAHA>


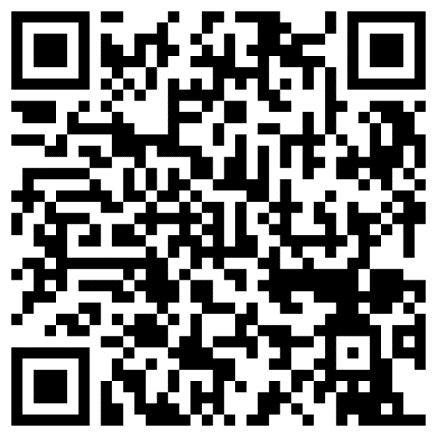


Please fill 2^nd^ questionnaire out within 72 hours of treatment:

<https://forms.gle/Z1rG9K8jmU8UFt6d8>


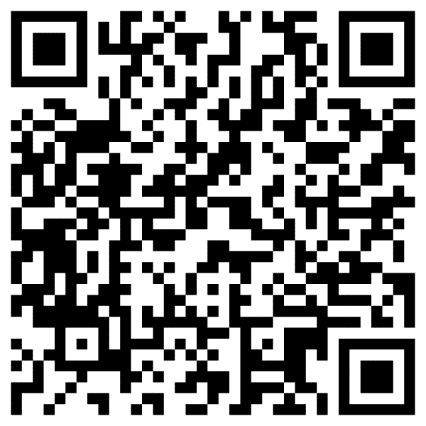


Follow up Questionnaire to be filled out at 2^nd^, 3^rd^, and 4^th^ appointments:

<https://forms.gle/EwQNwvbZd3dCzn5HA>


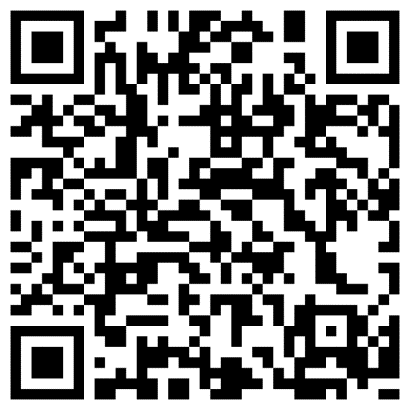

Supplement: Supplementary file 1 — Data S1. [file JOCD-23--s001.docx]
